# Primary progressive aphasia in sporadic and familial (type 8) amyotrophic lateral sclerosis ‐ A longitudinal study

**DOI:** 10.1002/alz.093166

**Published:** 2025-01-03

**Authors:** Caroline Martins de Araujo, Thais Helena Machado, Mariana Asmar Alencar Collares, Elisa de Paula França Resende, Leonardo Cruz de Souza

**Affiliations:** ^1^ Universidade Federal de Minas Gerais, Belo Horizonte‐MG, Minas Gerais Brazil; ^2^ Universidade Federal de Minas Gerais, Belo Horizonte, Minas Gerais Brazil; ^3^ UFMG, Belo Horizonte, Minas Gerais Brazil; ^4^ Universidade Federal de Minas Gerais, Belo Horizonte Brazil; ^5^ Departamento de Clínica Médica, Faculdade de Medicina, Universidade Federal de Minas Gerais, Belo Horizonte Brazil

## Abstract

**Background:**

Language is frequently affected in patients with sporadic amyotrophic lateral sclerosis (sALS), with reduced performance in naming, syntactic comprehension, grammatical expression, and orthographic processing. In a previous study, we demonstrated that the language profile of patients with familial type 8 ALS (ALS8), linked to p.P56S *VAPB* mutation, is similar to sALS. From a neuropsychological standpoint, there appears to be an overlap between primary progressive aphasia and ALS (PPA‐ALS) even in phases of the disease with little functional impairment. We aimed to investigate whether language decline has prognostic value in patients with ALS.

**Method:**

First, we performed a cross‐sectional evaluation. We included two groups of participants: 1) patients with sALS (n = 17); 2) patients with familial ALS8 (n = 22). The groups were matched for age, sex, and education level. All participants underwent a comprehensive language battery, including the Boston Diagnostic Aphasia Examination, the reduced Token Test, letter fluencies (FAS), categorical fluency (animals), word definition from the Cambridge Semantic Memory Research Battery, and a narrative discourse analysis. We also applied the Addenbrooke’s Cognitive Exam ‐ Revised Version (ACE‐R), the Hospital Anxiety and Depression Scale (HADS), and the ALS Functional Rating Scale‐Revised (ALSFRS‐R). The patients were reassessed 10‐65 months after the baseline visit with ALSFRS‐R, to follow up the progression rate of the disease.

**Result:**

We observed that the memory item of the ACE‐R test was able to predict functional declining in the fine motor domain in patients with sALS (F (1,16) = 19.443, R^2^ = 0.549, p<0,001).. The use of phonological cues in patients with ALS8 was able to predict global functional declining in this group (F (1,20) = 21.361, R^2^ = 0.516, p<0.001).

**Conclusion:**

Our data suggest that language decline may have prognostic value in cases of sALS and ALS8.

